# Database on the nonlinear optical properties of graphene based materials

**DOI:** 10.1016/j.dib.2019.105049

**Published:** 2019-12-31

**Authors:** Arpana Agrawal, Gyu-Chul Yi

**Affiliations:** Department of Physics and Astronomy, Institute of Applied Physics, Research Institute of Advanced Materials (RIAM), Seoul National University, Seoul 08826, South Korea

**Keywords:** Nonlinear absorption, Nonlinear refraction, Z-scan experiment, Graphene and its derivatives

## Abstract

The knowledge of optical nonlinearity is pre-requisite for the utility of the nonlinear optical (NLO) materials for optoelectronic device fabrication. Z-scan experimental technique based on the principles of spatial beam distortion, has been successfully employed for years to precisely investigate the NLO parameters. In the field of optical nonlinearity, graphene has proven itself as a strong candidate material owing to the possibility of strong light-matter interactions. A detailed comparison of the NLO properties of graphene and its derivatives (G/GDs) is crucial to identify and accelerate their utility for future flexible optoelectronic device applications. Herein, we share the experimental records of the optical nonlinearity in G/GDs, obtained from the well established Z-scan technique from the available literature, reported in the period from 2009 to 2019 and were extracted from the provided raw data [1]. The data sheet includes material composition, characteristics of the excitation laser source (operating wavelength, laser energy/power/intensity) and the NLO parameters (nonlinear absorption (NLA), nonlinear refraction (NLR), saturation intensity, optical limiting threshold). For practical use, they are tabulated in the present paper and will enable users to search the material data and filter down the set of desired materials using given parameters for their possible optoelectronic device applications. The data is related to the research article entitled “Unraveling absorptive and refractive optical nonlinearities in CVD grown graphene layers transferred onto a foreign quartz substrate” (Agrawal et al., 2019) [2].

**Table:** Specifications Table

Subject area	Materials Science
More specific subject area	Optical nonlinearity in two-dimensional (2D) graphene based materials
Type of data	Tables and figures
How data were acquired	Data is compiled for the experimentally obtained NLO parameters from the available literature reported in the period from 2009 to 2019.
Data format	Raw and analyzed
Parameters for data collection	Data compilation from available literature.
Description of data collection	Data is collected from the studies reported for the optical nonlinearity for graphene and its derivatives. The NLO parameters were obtained experimentally from the Z-scan technique.
Data source location	Seoul National University, Seoul, South Korea
Data accessibility	The raw data files are provided in the Mendeley Data, v2 https://doi.org/10.17632/mx4587rtsr.2 [1]. All the other data is with this article.
Related research article	Arpana Agrawal, Joon Young Park, Pratima Sen, Gyu-Chul Yi, Unraveling absorptive and refractive optical nonlinearities in CVD grown graphene layers transferred onto a foreign quartz substrate, Applied Surface Science, https://doi.org/10.1016/j.apsusc.2019.144392 (In press)

**Table:** 

**Value of the Data** • The data presented here is acquired using the Z-scan technique and covers the critical NLO properties of G/GDs from the previously published Z-scan experimental reports since 2009 until the end of 2019. • The database can be used to assess the potential of G/GDs as possible NLO materials. • The database will enable users to search the material data and filter down the set of desired materials using given parameters. • The database can be further used to accelerate the development in the field of graphene based materials for their possible optoelectronic applications.

## 1 Data

**Fig. 1:**
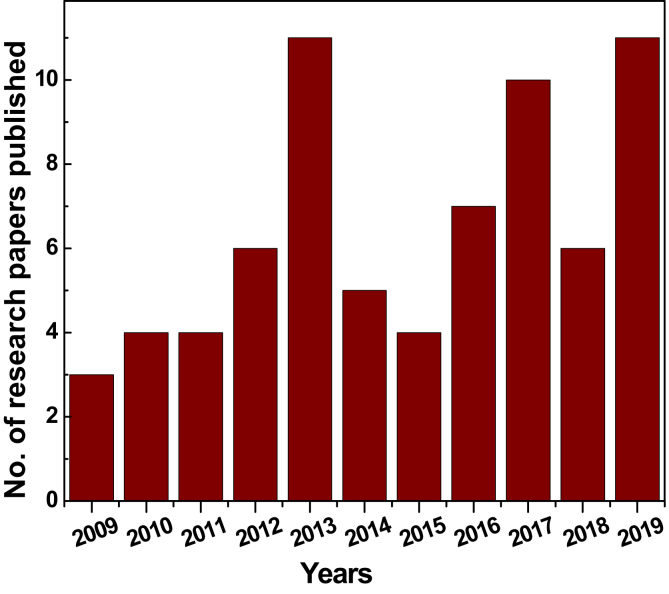
Histogram showing the number of research articles published from 2009 onwards in the field of experimental determination of NLO parameters of graphene based materials.

**Fig. 2:**
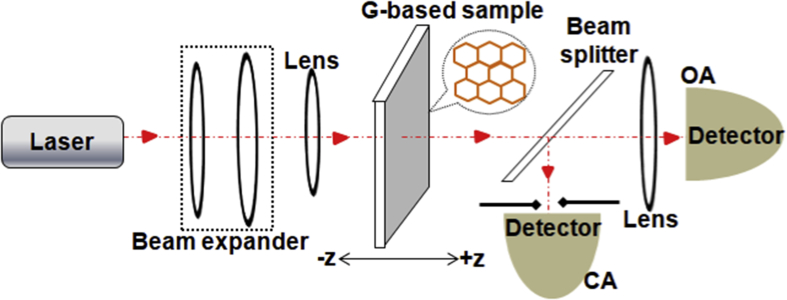
Schematic of the Z-scan experimental setup.

**Fig. 3:**
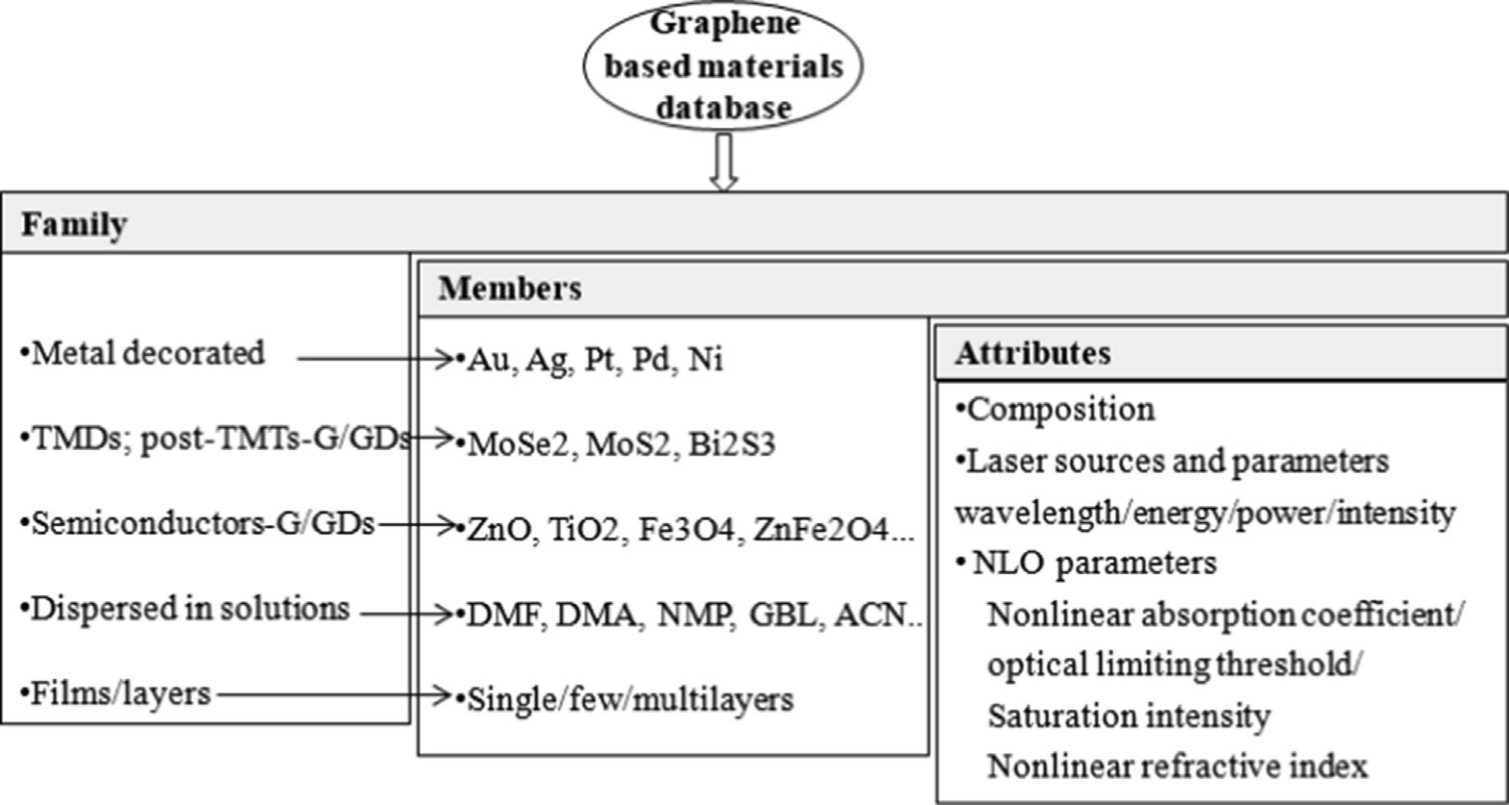
Tree-like classification of the graphene based materials database.

The database presented in this article describes a detailed comparison of the NLO properties of G/GDs, obtained experimentally from the Z-scan techniques, from the available literature reported in the period from 2009 to 2019. Fig. 1 (obtained from the raw data provided for the year-wise bifurcated research publications [1]) shows a histogram to graphically display the number of experimental research articles published from 2009 onwards, which clearly suggests the increasing interest of the scientific community to examine the optical nonlinearities in G/GDs. Fig. 2 depicts a schematic diagram for the Z-scan experimental setup, employed to investigate the optical nonlinearity. Fig. 3 illustrates a tree-like classification for the graphene based materials database, where the database is primarily classified in five families, including metal decorated-G/GDs, 2D transition metal dichalcogenides (TMDs) and post-transition metal trichalcogenides (TMTs)-G/GDs, semiconductor-graphene based materials, G/GDs dispersed in various solutions and single(SL)/few(FL)/multilayer(ML) graphene material based films. Each family has several members which are characterized by a set of attributes including: composition, laser parameters (operating excitation wavelength, laser energy/power/intensity) and experimentally derived NLO parameters (NLA, NLR, saturation intensity (Is), optical limiting threshold (F_th_)). Table 1 listed the NLO parameters of various graphene based materials and these entries makes up a material record which is highly valuable for the researchers interested in the optical nonlinearity in G/GDs from the experimental point of view.

**Table 1:** Database for the NLO parameters (NLA/NLR/Is/F_th_) investigated under various laser parameters (wavelength/laser power/energy/intensity) derived from Z-scan experimental studies for metal decorated-G/GDs, 2D-TMDs, post-TMTs-G/GDs, semiconductor-graphene based materials, G/GDs dispersed in various solutions and single/few/multilayer graphene material based films and many others. Parenthesis (column 2) indicates the reference number of published articles that are given in Ref. [1].

Material	Ref. [1]	Laser parameters	NLO parameters
Graphene and its derivatives decorated with various metals			
Au FG	(21)	514 nm	NLA = 128 cm/mW; NLR = − 0.292 cm^2^/MW
Pt NP/rGO	(25)	532 nm; 1 Hz; 4 ns; 50 μJ	NLA = 1.38 cm/GW
Ni NP/rGO	(25)	532 nm; 1 Hz; 4 ns; 50 μJ	NLA = 1.29 cm/GW
Pt–Ni NP/rGO	(25)	532 nm; 1 Hz; 4 ns; 50 μJ	NLA = 1.64 cm/GW
Pt–Ni cluster/rGO	(25)	532 nm; 1 Hz; 4 ns; 50 μJ	NLA = 1.98 cm/GW
GO-Ag	(30)	532 nm; 10 ns; 0.20 GW/cm^2^	NLA = 45.4 cm/GW
GO-Ag	(30)	532 nm; 10 ns; 0.16 GW/cm^2^	NLA = 39.7.4 cm/GW
GO-Ag	(30)	532 nm; 10 ns; 0.10 GW/cm^2^	NLA = 32.4 cm/GW
GO-Ag	(30)	532 nm; 10 ns; 0.08 GW/cm^2^	NLA = 30.0 cm/GW
NF-rGO/Ag-NPs(1 M)	(31)	532 nm; 5 ns	NLA = 13.9 m/GW
NF-rGO/Ag-NPs(1 M)	(31)	800 nm; 100 fs	NLA = 5.8 × 10^−15^ m/W
Au-NPs(16.55nm)/GO	(39)	532 nm; 180 mW	NLR = − 1.85 cm^2^/GW
Au-NPs(13.41 nm)/GO	(39)	532 nm; 180 mW	NLR = −2.7 cm^2^/GW
Au-NPs(9.52nm)/GO	(39)	532 nm; 180 mW	NLR = − 4.1 cm^2^/GW
Au-NPs(5.18nm)/GO	(39)	532 nm; 180 mW	NLR = − 5.8 cm^2^/GW
Pt/f-HEG	(49)	532 nm; 5 ns; 0.5 Hz	F_th_ = 13.7 J/cm^2^
Pd/f-HEG	(49)	532 nm; 5 ns; 0.5 Hz	F_th_ = 8.8 J/cm^2^
Pt/f-HEG	(49)	800 nm; 100 fs; 0.5 Hz	F_th_ = 1.8 J/cm^2^
Pd/f-HEG	(49)	800 nm; 100 fs; 0.5 Hz	F_th_ = 1.5 J/cm^2^
Ag NPs/fG composite	(58)	532 nm; 40 ps; 10 Hz	NLA = 812 cm/GW; Is = 3.7 GW/cm^2^
Ag NPs/fG composite	(58)	1064 nm; 40 ps; 10 Hz	NLA = 600 cm/GW
Ag NPs/rGO	(12)	–	Is = 18.5 MW/cm^2^; NLR = −1.1 × 10^−12^ m^2^/W
2D TMDs/graphene derivatives, post-TMTs-G/GDs			
MoSe2/G (Rt = 6h)	(7)	532 nm; 30 ps; 10 Hz; 6.6 GW/cm^2^	NLA = − 6.46 × 10^−12^ m/W; NLR = 1.54 × 10^−11^ esu
MoSe2/G (Rt = 12h)	(7)	532 nm; 30 ps; 10 Hz; 6.6 GW/cm^2^	NLA = −6.12 × 10^−12^ m/W; NLR = 1.35 × 10^−11^ esu
MoSe2/G (Rt = 18h)	(7)	532 nm; 30 ps; 10 Hz; 6.6 GW/cm^2^	NLA = −3.90 × 10^−12^ m/W; NLR = 1.18 × 10^−11^ esu
MoSe2/G (Rt = 24h)	(7)	532 nm; 30 ps; 10 Hz; 6.6 GW/cm^2^	NLA = −2.30 × 10^−12^ m/W; NLR = 1.14 × 10^−11^ esu
MoS2/G	(27)	800 nm; 1 Hz	NLA ∼ −1217.8 cm/GW
MoS2/G	(38)	400 nm; 100fs	Is = 1.427 GW/cm^2^
MoS2/G	(38)	800 nm; 100 fs	Is = 2.02 GW/cm^2^
MoS2/G	(38)	1562.6 nm; 565 fs	Is = 2.44 mW/cm^2^
G/MoS2/PMMA	(51)	532 nm; 6 ns; 1Hz; 66 μJ	NLA = 2110 cm/GW
Bi2S3/rGO (15mg)	(8)	532 nm; 30 ps; 10 Hz; 4.5 GW/cm^2^	NLA = 2.29 × 10^−11^ m/W; NLR = 1.65 × 10^−11^ esu
Bi2S3/rGO (30mg)	(8)	532 nm; 30 ps; 10 Hz; 4.5 GW/cm^2^	NLA = 2.48 × 10^−11^ m/W; NLR = 4.03 × 10^−11^ esu
Bi2S3/rGO (45mg)	(8)	532 nm; 30 ps; 10 Hz; 4.5 GW/cm^2^	NLA = 2.28 × 10^−11^ m/W; NLR = 3.48 × 10^−11^ esu
Bi2S3/rGO (60 mg)	(8)	532 nm; 30 ps; 10 Hz; 4.5 GW/cm^2^	NLA = 1.77 × 10^−11^ m/W; NLR = 1.81 × 10^−11^ esu
Semiconductor and graphene derivatives			
CdSe-rGO	(4)	532 nm; 30 ps; 10 Hz	NLA = 224.42 cm/GW; NLR = 48.90 × 10^−11^ esu
rGO-PbS QDs	(36)	532 nm; 4 ns; 10 Hz; 25 μJ	NLA = 7.9 × 10^−10^ m/W
G/CdS/PMMA	(50)	532 nm; 6 ns; 1 Hz	NLA = 797 cm/GW
GNS–CdS QDs in DMF	(67)	532 nm; 8 ns; 1 Hz; 250 μJ	NLA = 1.37 × 10^−13^ cm/W; F_th_ ∼0.88 J/cm^2^
GNS–CdS QDs in DMF	(67)	1064 nm; 8 ns; 1 Hz; 250 μJ	NLA = 0.55 × 10^−13^ cm/W; F_th_ > 6.4 J/cm^2^
TiO2/rGO (0.25 g)	(35)	532 nm; 4 ns	NLA = 6.0 × 10^−10^ m/W
(G/ZnO)3.9/PMMA	(40)	532 nm; 6 ns; 1 Hz	NLA = 415 cm/GW
G/ZnO)7.8/PMMA	(40)	532 nm; 6 ns; 1 Hz	NLA = 1530 cm/GW
GO–Fe3O4	(62)	532 nm; 5 ns; 10 Hz	NLA = 26 cm/GW; NLR = 2.83 × 10^−13^ cm^2^/W
S-rGO(10 mg)-ZnO	(46)	532 nm; 5 ns; 10 Hz	NLA = 5.8 cm/GW; Is = 1.5 GW/cm^2^
S-rGO(30 mg)-ZnO	(46)	532 nm; 5 ns; 10 Hz	NLA = 11 cm/GW; Is = 0.6 GW/cm^2^
H-rGO (10 mg) ZnO	(46)	532 nm; 5 ns; 10 Hz	NLA = 7.5 cm/GW; Is = 0.95 GW/cm^2^
H-rGO (30 mg) ZnO	(46)	532 nm; 5 ns; 10 Hz	NLA = 15 cm/GW; Is = 1.8 GW/cm^2^
GO–Fe3O4	(68)	532 nm; 5 ns	RSA: enhanced by Fe3O4
ZnFe2O4-rGO (40 wt%)	(24)	532 nm; 5 ns; 10 Hz; 100 μJ	NLA = 1.26 × 10^−10^ m/W; NLR = 1.29 × 10^−38^ esu
ZnFe2O4-rGO (25 wt%)	(24)	532 nm; 5 ns; 10 Hz; 100 μJ	NLA = 1.56 × 10^−10^ m/W; NLR = 1.33 × 10^−38^ esu
ZnFe2O4-rGO (15 wt%)	(24)	532 nm; 5 ns; 10 Hz; 100 μJ	NLA = 1.98 × 10^−10^ m/W; NLR = 2.50 × 10^−38^ esu
MgO-GO (T = 60 ^o^C)	(22)	532 nm; 10 ns; 200 Hz	NLA = 9.7 × 10^−8^ cm/W; NLR = −1.95 × 10^−12^ cm^2^/W
MgO-GO (T=180 ^o^C)	(22)	532 nm; 10 ns; 200 Hz	NLA = 1.5 × 10^−7^cm/W; NLR = −2.3 × 10^−12^ cm^2^/W
MgO-GO (T=210 ^o^C)	(22)	532 nm; 10 ns; 200 Hz	NLA = 1.8 × 10^−7^cm/W; NLR = −2.7 × 10^−12^ cm^2^/W
G/GDs dispersed in various solutions			
GO in DMF	(69)	532 nm; 5 ns	NLA = 5.6 × 10^−8^ cm/W; Is = 1.2 × 10^8^ W/cm^2^
GO in DMF	(69)	532 nm; 35 ps	NLA = 2.2 cm/GW; Is = 2.1 GW/cm^2^
G in DMA	(70)	532 nm; 6 ns; 10 Hz	F_th_ = 2 J/cm^2^
G in NMP	(70)	532 nm; 6 ns; 10 Hz	F_th_ ∼ 2.5 J/cm^2^
G in GBL	(70)	532 nm; 6 ns; 10 Hz	F_th_ ∼ 3 J/cm^2^
G in DMA	(70)	1064 nm; 6 ns; 10 Hz	F_th_ = 4.2 J/cm^2^
G in NMP	(70)	1064 nm; 6 ns; 10 Hz	F_th_ ∼ 7.8 J/cm^2^
G in GBL	(70)	1064 nm; 6 ns; 10 Hz	F_th_ ∼ 10 J/cm^2^
G sheet in water	(65)	532 nm; 8 ns	F_th_ = 0.25/3.2 (mJ/J cm^−2^)
G sheet in DMF	(65)	532 nm; 8 ns	F_th_ = 0.15/1.9 (mJ/J cm^−2^)
G sheet in THF	(65)	532 nm; 8 ns	F_th_ = 0.097/1.2 (mJ/J cm^−2^)
G sheet in ACN	(65)	532 nm; 8 ns	F_th_ = 0.11/1.4 (mJ/J cm^−2^)
GO in DMF	(63)	532 nm; 6 ns; 10 Hz	NLA = 30.22 cm/GW
GO in DMF	(63)	1064 nm; 6 ns; 10 Hz	NLA = 6.19 cm/GW
GO/water	(59)	532 nm; 5 ns	NLA = 0.35 nm/W
G in alcohol	(56)	800 nm; 50fs; 1kHz; 46 GW/cm^2^	NLA = 1.96 × 10^−2^ cm/GW
GO in water	(56)	800 nm; 50fs; 1kHz; 46 GW/cm^2^	NLA = −6.84 × 10^−3^cm/GW
GO NPs in water	(57)	810 nm; 150 fs; 80 MHz	NLA (2PA) ∼ 0.045 cm/GW (740 nm) to ∼ 0.023 cm/GW (820–850 nm)
GO NPs in water	(57)	1260 nm; 130 fs; 1 kHz	NLA = 1.84 × 10^−5^cm^3^ GW^−2^ (1200 nm) tõ 0.6 × 10^−5^ cm^3^ GW^−2^ (1260–1320 nm)
GF in DMF	(53)	532 nm; 4 ns	NLA = 20.71 cm/GW
GF in DMF	(53)	1064 nm; 4 ns	NLA = 12.53 cm/GW
GO in DMF	(54)	800 nm; 120 fs; 1 kHz; 82.1 GW/cm^2^	NLA = 2.5 × 10^−11^ cm/W; NLR = −5.3 × 10^−16^ cm^2^/W
G in DMF	(47)	532 nm; 6 ns; 2 Hz; 57∼166 nJ	NLA = 2.89 ± 0.15 cm/GW
G in NMP	(47)	532 nm; 6 ns; 2 Hz; 57∼166 nJ	NLA = 2.6 ± 0.13 cm/GW
G in SC	(47)	532 nm; 6 ns; 2 Hz; 57∼166 nJ	NLA = 2.05 ± 0.10 cm/GW
GO sheets in water	(44)	532 nm; 4 ns	NLA = 130.10 cm/GW
GO sheets in water	(44)	1064 nm; 4 ns	NLA = 20.01 cm/GW
GO in DMSO	(43)	532 nm; 4 ns; 10 Hz; 0.43 J/cm^2^	NLA = 50cm/GW
SLGO in water	(28)	532 nm; 4 ns	NLA = 108.4 cm/GW
FLGO in DMF	(28)	532 nm; 4 ns	NLA = 220 cm/GW
SLGO in water	(28)	532 nm; 35 ps	NLA = 2.08 cm/GW
GO/Water (% V) 0.82	(16)	534 nm	NLR = −7.060 cm^2^/GW
GO/Water (% V) 1.63	(16)	534 nm	NLR = −7.876 cm^2^/GW
GO/Water (% V) 2.43	(16)	534 nm	NLR = −8.679 cm^2^/GW
GO/Water (% V) 3.22	(16)	534 nm	NLR = −9.381 cm^2^/GW
GO/Water (% V) 4.00	(16)	534 nm	NLR = −9.971 cm^2^/GW
G NSs in water (hydrazine hydrate=5ml)	(13)	532 nm; 30 ps; 1 Hz	NLA = 1.11 cm/GW
G NSs in water (15 ml)	(13)	532 nm; 30 ps; 1 Hz	NLA = 3.67 cm/GW
G NSs in water (25 ml)	(13)	532 nm; 30 ps; 1 Hz	NLA = 4.32 cm/GW
GF in DMF	(28)	532 nm; 4 ns	NLA = 12.7 cm/GW; NLR = 14.7 × 10^−18^ m^2^/W
f-GF in water	(28)	532 nm; 4 ns	NLR = −34.2 × 10^−18^ m^2^/W
f-GF in water	(28)	532 nm; 35 ps	NLA = 0.8 cm/GW; NLR = −1.2 × 10^−18^ m^2^/W
GF in DMF	(28)	532 nm; 35 ps	NLR = 0.16 × 10^−18^ m^2^/W
GF in DMF	(53)	532 nm; 4 ns	NLA = 20.71 cm/GW
GF in DMF	(53)	1064nm; 4 ns	NLA = 12.53 cm/GW
F−GO/water	(59)	532 nm; 5 ns	NLA = 1.40 nm/W
HF−GO/NMP	(59)	532 nm; 5 ns	NLA = 0.7 nm/W
GO–ZnPc in DMSO	(43)	532 nm; 4 ns; 10 Hz; 0.43 J/cm^2^	NLA = 300 cm/GW
r-GO–ZnPc in DMSO	(43)	532 nm; 4 ns; 0.43 J/cm^2^	NLA = 1500 cm/GW
G-Cu porphyrin in DMF	(61)	532 nm; 6 ns; 10 Hz	NLA = 3570 cm/GW
GO–PcZn in DMF	(63)	532 nm; 6 ns; 10 Hz	NLA = 51.16 cm/GW
GO–PcZn in DMF	(63)	1064 nm; 6 ns; 10 Hz	NLA = 31.04 cm/GW
FGO2 (3–5 layers)/DMF	(64)	800 nm; 120 fs; 1 kHz; 303 GW/cm^2^	NLA = 1 × 10^−11^ cm/W
Graphene, graphene oxide, reduced graphene oxide films/layers			
G/quartz	(6)	690–1050 nm; 80MHz; 100 fs	NLR = 9.07 × 10^−9^ to 1.76 × 10^−8^ cm^2^/W
MLG/quartz	(5)	633 nm; 20 mW	NLA = 15.4 × 10^−3^ m/W; NLR = −14.5 m^2^/GW
Annealed MLG/quartz (250^o^C)	(5)	633 nm; 20 mW	NLA = 4.2 × 10^−3^ m/W; NLR = −1.4 m^2^/GW
rGO/PMMA	(17)	532 nm; 2 Hz; 300 μJ	NLA = 129.01 cm/GW
PFTP-rGO/PMMA	(17)	532 nm; 2 Hz; 300 μJ	NLA = 215.77 cm/GW
Annealed PFTP-rGO/PMMA	(17)	532 nm; 2 Hz; 300 μJ	NLA = 296.79 cm/GW
rGO/PMMA	(17)	1064 nm; 2 Hz; 300 μJ	NLA = 148.42 cm/GW
PFTP-rGO/PMMA film	(17)	1064 nm; 2 Hz; 300 μJ	NLA = 300.13 cm/GW
Annealed PFTP-rGO/PMMA film	(17)	1064 nm; 2 Hz; 300 μJ	NLA = 369.89 cm/GW
MLG (5–7 layers)	(32)	1150 nm; 100 fs; 1 kHz; 22GW/cm^2^	NLA = 0.38 × 10^4^ cm/W; NLR = −0.55
MLG (5–7 layers)	(32)	1550 nm; 100 fs; 1 kHz; 22GW/cm^2^	NLA = 0.9 × 10^4^ cm/W; NLR = −0.8
MLG (5–7 layers)	(32)	1900 nm; 100 fs; 1 kHz; 22GW/cm^2^	NLA = 1.5 × 10^4^ cm/W; NLR = −1.4
MLG (5–7 layers)	(32)	2400 nm; 100 fs; 1 kHz; 22GW/cm^2^	NLA = 1.9 × 10^4^ cm/W; NLR = −2.5
electrochemically derived GO film	(33)	800 nm; 10 kHz; 85 fs; 80mJ/cm^2^	NLA = −2 cm/GW; NLR = 3.63 cm^2^/GW
electrochemically derived GO film	(33)	800 nm; 10 kHz; 85 fs; 100mJ/cm^2^	NLA = +3 cm/GW; NLR = 2.82 cm^2^/GW
electrochemically derived GO film	(33)	800 nm; 10 kHz; 85 fs; 200 mJ/cm^2^	NLA = +5 cm/GW; NLR = 1.91 cm^2^/GW
electrochemically derived GO film	(33)	800 nm; 10 kHz; 85 fs; 400mJ/cm^2^	NLA = +7 cm/GW; NLR = 0.57 cm^2^/GW
GO films/glass	(60)	400nm; 100 fs; 1 kHz	NLA ∼ 41 000 cm/GW
GO films/glass	(60)	800 nm; 100 fs; 1 kHz	NLA(2PA) ∼31 cm/GW; NLA(3PA) ∼ 0.47cm^3^-GW^−2^
G layers/quartz	(55)	1550 nm; 3.8 ps; 10 MHz	NLR ≃ 10^−7^ cm^2^/W
GO thin film	(48)	800 nm; 100 fs; 70 GW/cm^2^	NLA = 0.47 × 10^−18^ cm^3^/GW^2^
G/quartz	(45)	733 nm; 100 fs; 80 MHz; 94 GW/cm^2^	NLA = 6 cm/MW; NLR = 1.4 cm^2^/GW
GO films	(26)	1560 nm; 67 fs; 20 MHz	NLA ∼ 10^3^ cm/GW; NLR = 0.45 cm^2^/GW
SGO	(64)	800 nm; 120 fs; 1 kHz; 303 GW/cm^2^	NLA = 4 × 10^−11^ cm/W
FGO1 (3–4 layers)	(64)	800 nm; 120 fs; 1 kHz; 303 GW/cm^2^	NLA = 3 × 10^−11^ cm/W
G/PMMA	(50)	532 nm; 6 ns; 1 Hz	NLA = 242 cm/GW
Others			
GO	(1)	532nm; 30 mW	NLA = 20.69 × 10^−5^ cm/W; NLR = 9.68 cm^2^/GW
GO	(1)	532nm; 40 mW	NLA = 138 cm/MW; NLR = 10.64 cm^2^/GW
GO	(1)	532nm; 50 mW	NLA = 43.6 cm/MW; NLR = 12.54 cm^2^/GW
rGO	(2)	532 nm	NLA = −0.01 × 10^−4^ cm/W; NLR = −8.83 × 10^−8^ cm^2^/W
r-GO (25%)-PANI	(2)	532 nm	NLA = −0.03 × 10^−4^ cm/W; NLR = −11.28 × 10^−8^ cm^2^/W
r-GO (50%)-PANI	(2)	532 nm	NLA = −0.09 × 10^−4^ cm/W; NLR = −13.43 × 10^−8^ cm^2^/W
rGO(75%)-PANI	(2)	532 nm	NLA = −0.04 × 10^−4^ cm/W; NLR = −12.06 × 10^−8^ cm^2^/W
GO	(3)	532 nm; 150 mW	NLR = 1.2 cm^2^/GW
GO–SiO2 (0.5:1)	(3)	532 nm; 150 mW	NLR = 6.2 cm^2^/GW
GO–SiO2 (1: 1)	(3)	532 nm; 150 mW	NLR = 6.8 cm^2^/GW
GO–SiO2 (2:1)	(3)	532 nm; 150 mW	NLR = 8.5 cm^2^/GW
rGO	(4)	532 nm; 30 ps; 10 Hz	NLA = 0.75 cm/GW; NLR = 0.21 × 10^−11^ esu
rGO	(8)	532 nm; 30 ps; 10 Hz; 4.5 GW/cm^2^	NLA = −1.23 × 10^−11^ m/W; NLR = 0.71 × 10^−11^ esu
GO	(9)	527 nm; 1 kHz; 150 ns; 28MW/cm^2^	NLA = 15.14 cm/GW
f-GO/sol	(9)	527 nm; 1 kHz; 150 ns; 28MW/cm^2^	NLA = 53.10 cm/GW
GO	(9)	527 nm; 1 kHz; 150 ns; 56MW/cm^2^	NLA = 21.65 cm/GW
f-GO/sol	(9)	527 nm; 1 kHz; 150 ns; 56MW/cm^2^	NLA = 345.92 cm/GW
GO	(9)	527 nm; 1 kHz; 150 ns; 113 MW/cm^2^	NLA = 14.06 cm/GW
f-GO/sol	(9)	527 nm; 1 kHz; 150 ns; 113 MW/cm^2^	NLA = 378.45 cm/GW
ZnNcC4-GO	(10)	532nm; 4ns; 10 Hz; 0.56 J/cm^2^	NLA = 220 cm/GW
ZnNcC4-NGO	(10)	532nm; 4ns; 10 Hz; 0.56 J/cm^2^	NLA = 450 cm/GW
ZnPcC4-NGO	(10)	532nm; 4ns; 10 Hz; 0.56 J/cm^2^	NLA = 360 cm/GW
GO	(11)	650 nm; 120 mW	NLA = 0.0808 cm/W; NLR = −2.36 × 10^−7^ cm^2^/W
rGO	(10)	650 nm; 120 mW	NLA = 0.0025 cm/W; NLR = −1.33 cm^2^/MW
rGO	(12)	Femtosecond laser	Is = 25.1 MW/cm^2^; NLR = −4.9 × 10^−13^ m^2^/W
GO	(14)	532 nm; 1 Hz; 4 ns; 8.53 GW/cm^2^	NLA = 1.68 × 10^−11^ cm/W
G-ZnO	(15)	1030 nm; 340 fs; 100 Hz; 18.7GW/cm^2^	NLA(2PA) = −78.6 × 10^−3^ cm/GW
G-ZnO	(15)	1030 nm; 340 fs; 100 Hz; 28.1GW/cm^2^	NLA(3PA) = 8.54 × 10^−3^ cm^3^-GW^−2^
G-ZnO	(15)	1030 nm; 340 fs; 100 Hz; 46.8GW/cm^2^	NLA(5PA) = 0.078 × 10^−3^ cm^7^-GW^−4^
h-BN NSs-GO (0.1mg/ml)	(18)	532 nm; 8 ns; 0.13 GW/cm^2^	NLA = 13.4 cm/GW
h-BN NSs-GO (0.1mg/ml)	(18)	532 nm; 8 ns; 0.16 GW/cm^2^	NLA = 17 cm/GW
h-BN NSs-GO (0.1mg/ml)	(18)	532 nm; 8 ns; 0.20 GW/cm^2^	NLA = 14.3 cm/GW; NLR = 2.58 × 10^−13^ cm^2^/W
GO	(19)	532 nm; 6 ns; 10 Hz; 150 μJ	NLA = 1.07 cm/GW
ZnP-GO	(19)	532 nm; 6 ns; 10 Hz; 150 μJ	NLA = 2.80 cm/GW
PF-GO	(19)	532 nm; 6 ns; 10 Hz; 150 μJ	NLA = 4.99 cm/GW
ZnP-rGO	(19)	532 nm; 6 ns; 10 Hz; 150 μJ	NLA = 6.58 cm/GW
PF-rGO	(19)	532 nm; 6 ns; 10 Hz; 150 μJ	NLA = 7.07 cm/GW
GO	(19)	1064 nm; 6 ns; 10 Hz; 1000μJ	NLA = 0.16 cm/GW
ZnP-GO	(19)	1064 nm; 6 ns; 10 Hz; 1000μJ	NLA = 0.23 cm/GW
PF-GO	(19)	1064 nm; 6 ns; 10 Hz; 1000μJ	NLA = 0.38 cm/GW
ZnP-rGO	(19)	1064 nm; 6 ns; 10 Hz; 1000μJ	NLA = 1.96 cm/GW
PF-rGO	(19)	1064 nm; 6 ns; 10 Hz; 1000μJ	NLA = 5.0 cm/GW
GO-X(=6, 8, 10, 12)	(20)	1064 nm; 4 ns	NLA = 45 cm/GW (GO-6) to 9.8 cm/GW (GO-12)
Y(80, 100, 120, 140, 160)-rGO-X(6)	(20)	1064 nm; 4 ns	NLA = 58 cm/GW (80-rGO-6) to 560 cm/GW (160-rGO-6)
Y(100)-rGO-X(6)	(20)	1064 nm; 4 ns	NLR = +1.87 × 10^−17^ m^2^/W
Y(120)-rGO-X(6)	(20)	1064 nm; 4 ns	NLR = +7.32 × 10^−17^ m^2^/W
Y(140)-rGO-X(6)	(20)	1064 nm; 4 ns	NLR = +6.23 × 10^−17^ m^2^/W
Y(160)-rGO-X(6)	(20)	1064 nm; 4 ns	NLR = +7.36 × 10^−17^ m^2^/W
Y(180)-rGO-X(6)	(20)	1064 nm; 4 ns	NLA = 490 cm/GW; NLR = +8.40 × 10^−17^ m^2^/W
NFG	(21)	514 nm	NLA = 1.58 × 10^−2^cm/W; NLR = −1.52 cm^2^/MW
FG	(21)	514 nm	NLA = 8.75 × 10^−2^cm/W; NLR = −0.53 cm^2^/MW
r-GO	(50)	532nm; 50 mW	NLA = −2.62 × 10^−4^ cm/W
GO	(35)	532 nm; 1 Hz; 4 ns; 50 μJ	NLA = 1.22 cm/GW
G	(62)	800 nm; 1 Hz	NLA ∼ −961.6 cm/GW
GO	(23)	532 nm; 50 mW	NLA = 6.1 × 10^−3^ cm/W; NLR = 4.0 × 10^−8^ cm^2^/W
NFG	(21)	514 nm	NLA = 1.58 × 10^−2^cm/W; NLR = −1.52 cm^2^/MW
FG	(21)	514 nm	NLA = 8.75 × 10^−2^cm/W; NLR = −0.53 cm^2^/MW
r-GO	(50)	532nm; 50 mW	NLA = −2.62 × 10^−4^ cm/W
GO	(35)	532 nm; 1 Hz; 4 ns; 50 μJ	NLA = 1.22 cm/GW
G	(62)	800 nm; 1 Hz	NLA ∼ −961.6 cm/GW
GO	(23)	532 nm; 50 mW	NLA = 6.1 × 10^−3^ cm/W; NLR = 4.0 × 10^−8^ cm^2^/W
ZnFe2O-(15%) rGO	(29)	532 nm; 50 mW	NLA = 6.5 × 10^−3^ cm/W; NLR = 4.7 × 10^−8^ cm^2^/W
ZnFe2O-(25%) rGO	(29)	532 nm; 50 mW	NLA = 6.3 × 10^−3^ cm/W; NLR = 4.5 × 10^−8^ cm^2^/W
ZnFe2O-(40%) rGO	(29)	532 nm; 50 mW	NLA = 6.1 × 10^−3^ cm/W; NLR = 4.5 × 10^−8^ cm^2^/W
GO	(29)	800 nm; 80MHz; 150 fs	NLA = 12.0 × 10^−12^ m/W; NLR = 14.7 × 10^−18^ m^2^/W
ZnFe2O-(15%) rGO	(29)	800 nm; 80MHz; 150 fs	NLA = 4.0 × 10^−12^ m/W; NLR = 4.2 × 10^−18^ m^2^/W
ZnFe2O-(25%) rGO	(29)	800 nm; 80MHz; 150 fs	NLA = 3.1 × 10^−12^ m/W; NLR = 2.8 × 10^−18^ m^2^/W
ZnFe2O-(40%) rGO	(29)	800 nm; 80MHz; 150 fs	NLA = 2.6 × 10^−12^ m/W; NLR = 1.5 × 10^−18^ m^2^/W
GO	(30)	532 nm; 10 ns; 0.20 GW/cm^2^	NLA = 17 cm/GW
GO	(30)	532 nm; 10 ns; 0.16 GW/cm^2^	NLA = 13.5 cm/GW
GO	(30)	532 nm; 10 ns; 0.10 GW/cm^2^	NLA = 12 cm/GW
GO	(30)	532 nm; 10 ns; 0.08 GW/cm^2^	NLA = 10.8 cm/GW
NF-rGO	(31)	532 nm; 5 ns	NLA = 2.8 × 10^−10^ m/W
NF-rGO	(31)	800 nm; 100 fs	NLA = 2.5 × 10^−15^ m/W
GO	(34)	532 nm; 4 ns; 10 Hz	NLA = 80 cm/GW
ZnPcC4 –GO	(34)	532 nm; 4 ns; 10 Hz	NLA = 200 cm/GW
NGO	(34)	532 nm; 4 ns; 10 Hz	NLA = 120 cm/GW
ZnPcC4 –NGO	(34)	532 nm; 4 ns; 10 Hz	NLA = 370 cm/GW
rGO,	(35)	532 nm; 4 ns	NLA = 3.0 × 10^−10^ m/W
GO	(36)	532 nm; 4 ns; 10 Hz; 25 μJ	NLA = 1.0 × 10^−10^ m/W
GO–Cz	(37)	532 nm	NLA = 58.56 cm/GW
GO–Cz	(37)	1064 nm	NLA = 23.08 cm/GW
G1.1/PMMA	(40)	532 nm; 6 ns; 1 Hz	NLA = 275 cm/GW
rGO	(41)	800 nm; 100 fs; 32 μJ/cm	NLA = 40000 cm/GW
rGO	(41)	800 nm; 100 fs; >50 μJ/cm	NLR ∼ 10 ^−9^ cm^2^/W
f-HEG	(42)	800 nm; 100 fs	F_th_ = 2.7 J/cm^2^
CuO/f-HEG	(42)	532 nm; 5 ns	NLA = 10^−10^ m/W; F_th_ = 4.35 J/cm^2^
CuO/f-HEG	(42)	800 nm; 100 fs	NLA = 10^−14^ m/W; F_th_ = 1.43 J/cm^2^
GO	(46)	532 nm; 5 ns; 10 Hz	NLA = 5.4 cm/GW; Is = 1.7 GW/cm^2^
f-HEG	(49)	532 nm; 5 ns; 0.5 Hz	NLA ∼ 10^−11^ to 10^−10^ m/W
f-HEG	(49)	800 nm; 100 fs; 0.5 Hz	NLA ∼ 10^−15^ m/W; F_th_ = 2.7 J/cm^2^
GO-NH2–Pani hybrid	(52)	532 nm; 5 ns; 10 Hz; 35 μJ	NLA = 8.5 cm/GW; Is = 0.8 GW/cm^2^
GO	(54)	800 nm; 120 fs; 1 kHz; 82.1 GW/cm^2^	NLA = 1.6 × 10^−11^ cm/W; NLR = −1.1 × 10^−15^ cm^2^/W
fG	(58)	1064 nm; 40 ps; 10 Hz	NLA = 280 ± 18 cm∕GW; Is = 8 ± 0.2 GW∕cm^2^
G	(61)	532 nm; 6 ns; 10 Hz	NLA = 132cm/GW
G-Zn porphyrin	(61)	532 nm; 6 ns; 10 Hz	NLA = 4720 cm/GW
GO	(62)	532 nm; 5 ns; 10 Hz	NLA = 7.8 cm/GW; NLR = 9.74 × 10^−14^ cm^2^/W
GO	(65)	532 nm	NLA = 2.8 ± 0.1cm/GW
GONSs	(66)	532 nm; 250μJ/pulse	NLA = 0.29 × 10^−13^ cm/W; F_th_ > 3J/cm^2^
GNSs	(66)	532 nm; 250μJ/pulse	NLA = 1.36 × 10^−13^ cm/W; F_th_ = 0.5J/cm^2^
GONRs	(66)	532 nm; 250μJ/pulse	NLA = 1.09 × 10^−13^ cm/W; F_th_ = 1J/cm^2^
GNRs	(66)	532 nm; 250μJ/pulse	NLA = 1.26 × 10^−13^ cm/W; F_th_ = 0.7 J/cm^2^
GONSs	(66)	1064 nm; 250μJ/pulse	NLA = 0.12 × 10^−13^ cm/W
GNSs	(66)	1064 nm; 250μJ/pulse	NLA = 1.16 × 10^−13^ cm/W; F_th_ = 6.3J/cm^2^
GONRs	(66)	1064 nm; 250μJ/pulse	NLA = 1.40 × 10^−13^ cm/W; F_th_ = 4.0 J/cm^2^
GNRs	(66)	1064 nm; 250μJ/pulse	NLA = 1.6 × 10^−13^ cm/W; F_th_ = 3.4 J/cm^2^
Hydrothermally r-GO	(71)	1560 nm; 100 MHz; CW	Transmittance ∼57%; P_out_/P_in_ = 0.49
Hydrothermally r-GO	(71)	1560 nm; 100 MHz; 5 ns	Transmittance∼12%; P_out_/P_in_ = 0.11

**Abbreviations:** G: Graphene, GO: Graphene-oxide, rGO: reduced GO, *NF-rGO: non-covalent functionalized r-GO, NPs: Nanoparticles, NSs: Nanosheets, NRs: Nanorods, HEG:* hydrogen exfoliated G, f-HEG: functionalized HEG, PMMA: poly(methylmethacrylate), QDs: Quantum dots, DMF: N,N-dimethylformamide, S-rGO-ZnO: ZnO decorated GO deoxygenated in alkaline medium, H-rGOZnO: hydrothermally reduced GO decorated with ZnO, NMP: N-methyl-2-pyrrolidone, DMA: N,N-dimethylacetamide, GBL: g-butyrolactone, ACN: acetonitrile, THF: tetrahydrofuran, SC: sodium cholate, DMSO: Dimethyl sulfoxide, GF: G-fluoride, f-GF: non-covalent functionalized GF, F-GO: fluorinated GO, HF-GO: highly fluorinated GO, ZnPc: triethyleneglycol-substituted Zn(II) phthalocyanine, PcZn: zinc phthalocyanine, PFTP: poly[(9,9-dihexyl-9Hfluorene)- alt-(1,1,2,2-tetraphenylethene)], PANI: polyaniline, f-GO/sol: functionalized GO in an organosiloxane matrix, ZnNcC4-GO: tetracarboxylic zinc naphthalocyanine-GO, ZnNcC4-NGO: tetracarboxylic zinc naphthalocyanine-amino GO, PcZn: Zinc phthalocyanine.

## 2 Experimental design, materials, and methods

For all the literature presented in this article, the data was acquired from the well established Z-scan technique, pioneered by Sheik Bahae et al. [[3], [4], [5]], and has also been extensively described elsewhere [6,7]. Briefly, the NLO is the optics of light where NLA and NLR becomes intensity dependent. For this, a high power/intense laser light source is irradiated on the NLO material and the transmitted signal from the material was recorded as a function of intensity, varied by translating the sample through the focal plane of a tight focussing lens along its propagation (z) axis (Fig. 2). Z-scan experiment can be formed in two geometries: (i) open aperture (OA) geometry for extracting NLA, where all the transmitted light was collected and focused onto the detector using another lens, and (ii) closed aperture (CA) geometry where only the on-axis beam is allowed to be collected at the detector for the determination of NLR.

It is noteworthy to mention here that graphene has attracted much attention after Andre Geim and Kostya Novoselov were awarded the 2010 Nobel Prize in Physics “for groundbreaking experiments regarding the 2D material graphene”. After that, this material and their derivatives decorated with other materials *viz;* decorated with metals, TMDs, TMTs, semiconductors has been extensively investigated for various properties (Fig. 3). This material has also gained tremendous interest in the field of flexible optoelectronic device applications because of the possibility of strong light-matter interactions and flexible nature.

Although, there are a number of theoretical as well as experimental reports published in the field of optical nonlinearities in G/GDs. Herein, only the experimentally derived optical nonlinearity employing Z-scan technique has been listed (Table 1). Usually, OA experimental curve/result exhibits either a sharp peak or valley at the focal plane of the tight focussing lens as a consequence of saturable absorption (SA) or reverse saturation absorption (RSA), respectively. However, in graphene based materials, a combination of SA and RSA has also been observed where the transmittance from the material is found to increase at both the ± z-direction with respect to the focal plane, followed by a dip at the focus [2]. On the other hand, under CA geometry, the far-field on-axis normalized transmittance as a function of traversed distance exhibits a peak followed by a valley or vice versa. The prefocal peak (valley) and postfocal valley (peak) in the experimentally obtained curve indicates the negative (positive) sign of the NLR coefficient. These parameters were obtained from the best possible theoretical fitting to the experimental Z-scan profile depending upon the property of the grown material. The theoretical fitting of the experimental Z-scan profile is based on various phenomenon including; multi-photon absorption (n-PA, where integer n > 1), SA, RSA, NLO scattering, thermal lensing or a combination of these effects. Further details regarding the functions used for the fitting of the experimental data curves are given elsewhere [[2], [3], [4], [5], [6], [7]]. This work reflects the state-of-the-art of the novel graphene material and the NLO properties are not equally renowned for every derivative due to the lack of literature data. In view of this, listing of these entries (Table 1) makes up a material record which precisely represents the material property and will enable users to search the material data and filter down the set of desired materials using given parameters. The database was used by Agrawal et al. [2].
